# *OPA1* Dominant Optic Atrophy: Pathogenesis and Therapeutic Targets

**DOI:** 10.1097/WNO.0000000000001830

**Published:** 2023-04-19

**Authors:** David C. S. Wong, Joshua P. Harvey, Neringa Jurkute, Sara M. Thomasy, Mariya Moosajee, Patrick Yu-Wai-Man, Michael J. Gilhooley

**Affiliations:** Department of Clinical Neurosciences (DCSW, PY-W-M), John van Geest Center for Brain Repair, University of Cambridge, Cambridge, United Kingdom; Cambridge Eye Unit (DCSW, PY-W-M), Addenbrooke's Hospital, Cambridge, United Kingdom; UCL Institute of Ophthalmology (JPH, NJ, MM, PY-W-M, MJG), London, United Kingdom; Moorfields Eye Hospital NHS Foundation Trust (JPH, NJ, MM, PY-W-M, MJG), London, United Kingdom; Department of Ophthalmology and Vision Science (SMT), School of Medicine, U.C. Davis, Sacramento, California; Department of Surgical and Radiological Sciences (SMT), School of Veterinary Medicine, U.C. Davis, California; Great Ormond Street Hospital (MM), London, United Kingdom; and The Francis Crick Institute (MM), London, United Kingdom.

Dominant optic atrophy (DOA, OMIM 165500) is the most common inherited optic neuropathy (1), with prevalence estimates of 1:12,000 in Denmark (2) and 1:25,000 in North East England (3). Inherited optic neuropathies carry a poor visual prognosis and have a significant detrimental impact on the quality of life, with high rates of psychological distress and great societal costs (4–7).

DOA was first described in a British family by Batten in the 19th century (8) and confirmed in larger studies of German families by Jaeger (9) and of Danish families by Kjer (10). These studies demonstrated an autosomal dominant inheritance of isolated optic atrophy, which was clinically and genetically distinct to the maternally inherited Leber hereditary optic neuropathy (LHON, OMIM 535000), (11). Patients with DOA typically suffer an insidious, progressive, and bilateral visual loss starting in early childhood (12).

Fundus examination in DOA typically shows temporal pallor of the optic discs (Fig. 1A), corresponding with reduced retinal nerve fiber layer (RNFL) thickness of the papillomacular bundle visible on optical coherence tomography (OCT) imaging (Fig. 1B) (13). OCT angiography shows reduced blood flow in the temporal region of the optic disc (14–16). Macular OCT demonstrates thinning of the ganglion cell layer (Fig. 1C) (17), and perimetry usually shows bilateral central or centrocaecal scotoma (Fig. 1D). These clinical features illustrate that the loss of retinal ganglion cells (RGCs), particularly in the papillomacular bundle, is central to DOA pathogenesis. Approximately 20% of patients with DOA also have multisystemic features (“DOA plus”) such as sensorineural hearing loss, peripheral neuropathy, myopathy, spastic paraplegia, multiple sclerosis–like illness, and chronic progressive external ophthalmoplegia (18,19).

**FIG. 1.**
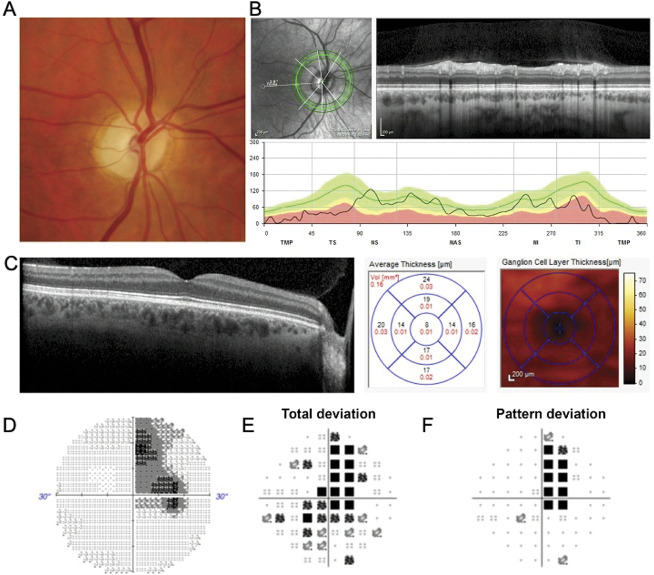
Typical ophthalmological features in a patient with *OPA1*-positive DOA. **A**. Color photograph of the right optic disc in a patient with DOA caused by an *OPA1* variant. The disc is diffusely pale with the pallor being more prominent temporally. **B**. Optical coherence tomography (OCT) imaging of the right optic disc demonstrating loss of the retinal nerve fiber layer particularly temporally. **C**. Macular OCT of right eye showing substantial RNFL thinning. **D**. Gray scale of perimetry obtained using a automated 30-2 SITA-Fast (Swedish Interactive Thresholding Algorithm) protocol demonstrating a dense asymmetric scotoma. **E, F**. Total deviation and pattern deviation of the same visual field. A dense superior scotoma is visible that extends into the inferior hemifield. DOA, Dominant Optic Atrophy; RNFL, Retinal Nerve Fibre Layer.

There is marked interfamilial and intrafamilial variability in the severity of the disease, with visual acuities ranging from normal to detection of hand movements only. Half of all DOA patients fail the driving standards and are registered legally blind (12). The source of this variability is not fully understood, and it is likely to be due to a combination of environmental, genetic, and other factors.

Although an increasing number of nuclear-encoded gene variants are being identified as the cause of DOA (20), 70%–90% of patients carry variants in the autosomal gene *OPA1* (3q29, OMIM 605290) (21,22). The *OPA1* gene is large, spanning >90 kbp of genomic DNA (23), and composed of 30 exons (Fig. 2). The transcript (NM_015560.2(OPA1)) is alternatively spliced into 8 different isoforms, and they are translated to Long- (120 kDa) and Short- (80 kDa) forms of OPA1 (24,25). The resulting OPA1 protein is a ubiquitously expressed dynamin-like GTPase protein that has a mitochondrial targeting sequence (MTS, Fig. 2), which facilitates mitochondrial import (26). Once incorporated into the inner mitochondrial membrane, OPA1 regulates a number of mitochondrial functions, including mitochondrial fusion, bioenergetics, mitophagy, and stabilization of the respiratory chain complexes (27). Therefore, DOA is considered to be a mitochondrial disease, despite the autosomal location of the most common causative gene (27). Other genes that cause DOA are also important for mitochondrial function; these genes have been described in recent reviews (20,27,28).

**FIG. 2.**
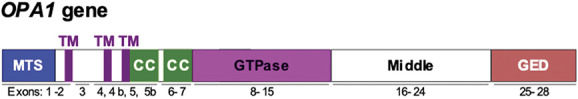
The *OPA1* gene. The human *OPA1* gene contains 30 exons. The translated protein consists of a mitochondrial targeting sequence (MTS), transmembrane domains (TM), coiled-coil domains (CC), GTPase, middle domain and a G-protein effector domain (GED).

There are more than 500 variants in the *OPA1* gene that are likely thought to be pathogenic (23,29). Of these, 28% are missense and mostly in the GTPase domain (29). A further 24% of these genes cause aberrant splicing: 22% cause frameshifts, 15% nonsense, and 7% structural variants (29). Most of these pathogenic variants lead to premature termination of translation, and they are therefore likely to be null alleles, which is consistent with the reduced OPA1 protein concentrations seen in patient-derived fibroblasts (30). Therefore, haploinsufficiency has been proposed to be the primary disease mechanism in *OPA1*-linked DOA (27). Haploinsufficiency (HI) is a mechanism of dominance where one normal copy of the protein is insufficient to maintain its normal cellular function (Fig. 3) (31). Hence, when there is one null allele, a disease phenotype results. By contrast, dominant-negative (DN) effects are cases where the abnormal protein interferes with the function of the wild-type protein (Fig. 3) (31). An increasing number of *OPA1* variants have been shown to result in DN effects. Predominantly, these are missense with a few isolated in-frame splice variants also described, both of which are associated with a more severe phenotype (32,33).

**FIG. 3.**
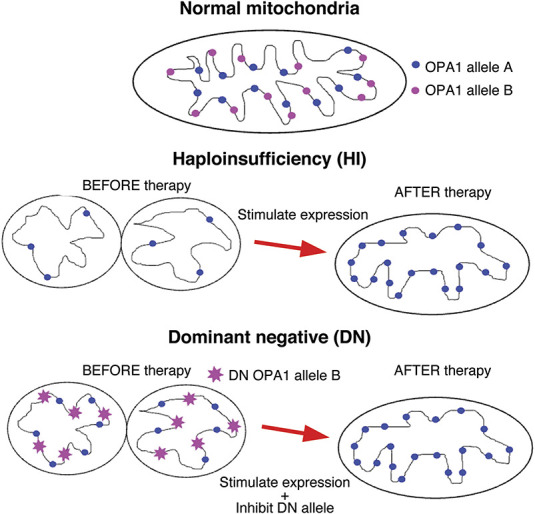
Haploinsufficiency and dominant negative effects. Haploinsufficiency (HI) occurs when one copy of the gene is a null allele, so it is not expressed at a sufficient level to facilitate normal function of the protein. In *OPA1* variants, this results in mitochondrial structural fragmentation, impaired adenosine triphosphate generation, and increased susceptibility to cell death. Therapeutically, this can be addressed by stimulating the expression of the remaining normal allele to protect or rescue mitochondrial function. Dominant negative (DN) effects are manifested when the product of an allele is a protein that interferes with the function of the normal allele. This may require targeted inhibition of the mutant allele in addition to stimulation of the remaining allele to restore normal cell physiology.

Understanding the molecular pathogenesis of *OPA1*-linked DOA may shed light on other diseases too. There is a growing body of evidence linking mitochondrial dysfunction with the neurodegenerative processes contributing to the development and progression of glaucoma (34), Parkinson's disease (35), and late-onset dementias (36), including Alzheimer's disease (37). Therefore, investigating the pathogenesis of DOA provides a powerful model system that has broader significance in advancing our understanding of other major neurodegenerative disorders. Characterization of the unique susceptibility of RGCs in DOA will also aid our understanding of other optic neuropathies, such as glaucoma, and guide the development of targeted therapies. In this perspective, we describe how the clinical presentation of patients with DOA sheds light on the molecular pathogenesis of this disease. We then outline how our understanding of this disease process is being enhanced by the use of increasingly sophisticated model systems, which are revealing potential therapeutic targets.

## PATHOGENESIS

Patients with DOA typically develop insidious bilateral visual loss, generalized dyschromatopsia, and temporal disc atrophy. This points toward a specific dysfunction of RGCs, particularly those constituting the papillomacular bundle. Despite *OPA1* being a ubiquitously expressed gene, RGCs appear particularly vulnerable to the effects of genetic variants. This may be because of the intrinsic structural properties of RGCs that make them susceptible to mitochondrial dysfunction. The axons of RGCs are very long—approximately 50 mm in humans (99% of their total cell volume), and they transmit around 15 action potentials per second (38). RGC axons run along the inner surface of the retina, through the lamina cribrosa, along the optic nerve, optic chiasm, and optic tract before synapsing in the lateral geniculate nucleus. The intraocular portion of the RGC is unmyelinated in order to facilitate transparency and retinal light penetration. Therefore, action potentials cannot rely on the energy-conserving saltatory conduction intraocularly, and so, a large amount of adenosine triphosphate (ATP) is required for the management of Na^+^ and K^+^ gradients. As a consequence, RGCs have a high metabolic requirement, and they are particularly dependent on mitochondrial aerobic respiration (39). RGC axons are myelinated after passing through the lamina cribrosa, so action potential propagation becomes less energetically demanding outside the eye. Consistent with this, mitochondria are distributed more densely in the intraocular portions of the RGC axons compared with the extraocular portions (40–42). At the lamina cribrosa, there is a transition zone between unmyelinated and myelinated axons; mitochondrial structure here appears to be very dynamic, involving both intramitochondrial and intermitochondrial membrane modifications (27).

The RGCs that make up the papillomacular bundle may be particularly vulnerable due to their more limited blood supply (14), increased exposure to oxidative stress generated by passage of light (43), and the relatively smaller caliber of the parvocellular RGC axons, which may physically restrict the transport and maintenance of mitochondria (44). Intrinsically photosensitive RGCs (ipRGCs) appear to be resistant to degeneration in this disease and in many animal models of neuronal damage (45–47). This is consistent with the finding that patients with DOA appear to have preserved physiological diurnal rhythms and pupillary light reflexes (45) because ipRGCs are crucial for circadian entrainment and nonvisual light perception (48–50). The reasons for this are not fully understood but may involve their relatively large diameter axons (44,51) or the expression of neuroprotective genes (52). The ipRGCs also uniquely express melanopsin as their light-sensitive pigment (53,54), but because ipRGCs are still spared in melanopsin knockout models of DOA, melanopsin expression in itself is less likely to be the cause of this resistance (55).

Most pathogenic *OPA1* variants are haploinsufficient, such as c.2708_2711del, which is the commonest (23). This means that the variant results in a null allele, and the one remaining unaffected allele is insufficient to functionally compensate for this loss. Consistent with this, experimentally knocking out or reducing expression of *OPA1* results in mitochondrial structural and functional abnormalities (56–62), whereas overexpression is protective against apoptotic insults (56,63). However, it is believed that supraphysiological overexpression of *OPA1* may be toxic (64).

Some *OPA1* variants exert pathogenic effects through dominant-negative mechanisms. For example, missense and possibly in-frame splicing variants such as c.2356-1G>T, which results in abnormal splicing, could manifest in dominant negativity by affecting OPA1 oligomerization (33). This is important because missense variants and those in the GTPase region of *OPA1* generally result in more severe DOA phenotypes and increased chance of extraocular features (19).

This distinction is important because different therapeutic approaches are needed for gene therapies (Fig. 3). An understanding of the transcriptional effects of variants is critical before the design of genetic modification strategies such as antisense oligonucleotides (ASOs). For instance, an ASO strategy aiming to upregulate wild-type (WT) transcript expression may not function in the context of a dominant negative variant, for which a knockdown of the disease-causing allele may be more appropriate. Gene therapies are therefore likely to be time consuming and costly to develop, with new variants having to be modeled to ascertain their transcriptional and translational impact. Critical to facilitating this modeling is the establishment of robust disease models, which will be addressed in the next section.

## MODELING SOLUTIONS

**FIG. 4.**
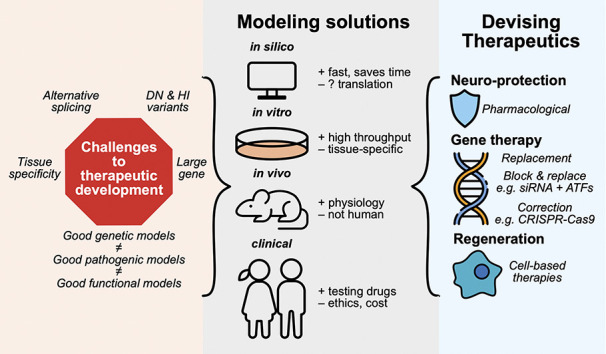
Challenges, models, and potential solutions. A therapeutic pipeline for DOA has proven challenging to advance due to the complexity and heterogeneity of the genetic basis of the disease. Various models have been used to better understand the underlying pathophysiology, ranging from in silico hypothesis generation to observational and interventional clinical trials in humans. Each model system has its own advantages and disadvantages, but when used together, exciting new therapeutics can be developed and validated. Current strategies involve the identification of neuroprotective agents that can prevent and restore vision in patients affected with DOA. Gene therapy techniques include the use of viral vectors to replace or correct genes, as well as block and replace gene expression modulation to address dominant negative effects. Finally, cell-based therapies may one day provide a way to regenerate RGCs that have already been lost, although significant hurdles remain. DOA, Dominant Optic Atrophy; RGC, Retinal Ganglion Cell.

Novel disease-causing variants in *OPA1* are constantly being reported, highlighting the marked genetic heterogeneity of this particular disease (23,29,65). To facilitate personalized genetic treatments, and to improve our understanding of DOA pathogenesis, a number of modeling solutions have been implemented (Fig. 4).

In silico models have been used to predict the pathogenicity of novel missense variants (23) and the effects of splice site variants (33,66). However, it remains challenging to determine the translational consequences of the predicted transcripts, and in vitro models provide an excellent way to validate these findings (66). As described above, the pathogenesis of DOA is centered around mitochondrial dysfunction, and so cell-based models can be ideal for exploring the haploinsufficiency and dominant-negative effects of *OPA1* variants.

Patient-derived fibroblasts and mouse embryonic fibroblasts provide convenient in vitro model systems to interrogate patient-specific *OPA1* variants and their impact on cellular physiology (33,67,68). However, this method may overlook tissue-specific actions of *OPA1*. Alternatively, somatic cells may be harvested from patients, for example, from the skin and then reprogrammed to achieve pluripotency. These induced pluripotent stem cells (iPSCs) may be used to create retina organoids (69–72) or specific RGC cultures (73–77). iPSC-derived RGCs also provide an excellent resource for drug screening and development of cellular therapies, in a patient-focused manner, avoiding the ethical issues surrounding the use of embryonic stem cells (78,79).

In vivo mouse models have also been used in the field of DOA research (27). Although the mouse *Opa1* genetic sequence is 96% similar to the human *OPA1*, there are important molecular differences. Humans have 8 isoforms of *OPA1*, whereas mice only have 4 (80). This is important because the correct balance of isoforms has been shown to be important for cellular function (25). This is of particular concern when developing gene therapies. Furthermore, mouse models demonstrate RGC loss primarily due to *OPA1* haploinsufficiency, and the insight into the dominant-negative effects of *OPA1* variants in humans remains limited (27).

One of the strengths of in vivo studies compared with in vitro studies is the ability to assess the effects of *OPA1* gene variants on visual function (81). There are well-established behavioral assays to assess vision in mice. However, these should be interpreted within the context of the mouse anatomy in that they do not have a fovea or papillomacular bundle (81), despite the fact that the mouse retina closely resembles the *peripheral* retina in primates (82). Indeed, it remains unclear whether mice have the same range of RGC subtypes as humans (81). Therefore, mouse models may not recapitulate all aspects of human DOA pathogenesis and visual function. Three major mouse models have been created to model *OPA1* DOA: *Opa1*^*+/del*^ (83), *Opa1*^*+/STOP*^ (84), and *Opa1*^*+/delTTAG*^ (85). Zebrafish and *Drosophila* are also useful model organisms that have been used to explore DOA pathogenesis (86–88), and the uses of each have been previously reviewed (27).

However, more recently rhesus macaques with a disease-causing variant in OPA1 have been identified at the California National Primate Research Center (89). In these nonhuman primates, peripapillary retinal nerve fiber layer thickness and retinal ganglion cell function were decreased, consistent with a DOA phenotype. Fortune et al (90) described idiopathic bilateral optic atrophy in rhesus macaques with RGC loss and dysfunction, but a genetic cause has not been identified. A similar syndrome exists in cynomolgus macaques (91), although this may be due to a variant in *WFS1*, which is the main causative gene in Wolfram syndrome, another form of inherited optic neuropathy (92). These spontaneous models recapitulate important clinical features of DOA and serve as valuable translational models to advancing novel therapeutic strategies for this disease.

## DEVISING THERAPEUTICS

Current and future treatment strategies for DOA can be divided into 3 broad categories: pharmacological neuroprotection, gene therapy, and cell-based regenerative therapies (Fig. 4). A major focus has been on gene therapy because this may halt neurodegeneration of RGCs in DOA. Pharmacological neuroprotection aims to prevent or slow damage to RGCs by providing antioxidants and other compounds to slow the progression of the disease. By contrast, cell-based regenerative therapies aim to replace RGCs that have already been damaged.

Pharmacological neuroprotective strategies that are being considered include idebenone, which is a synthetic analogue of coenzyme Q10. Idebenone was approved by the European Medicines Agency for the treatment of LHON after acute visual loss (93,94). The results of a trial using the mouse Opa1^+/STOP^ model showed some deleterious effects and a lack of efficacy (95), but a retrospective case series showed some stabilization and recovery of visual acuity (96). At the time of writing, there are further ongoing trials testing idebenone in patients with DOA (Table 1).

**TABLE 1.** Current clinical trials for DOA

Type	Agent	Title	Trial Number
Observational	N/A	Rare Disease Patient Registry & Natural History Study—Coordination of Rare Diseases at Sanford (CoRDS)	NCT01793168
Observational	N/A	Advanced Characterization of Autosomal Dominant Optic Atrophy	NCT01522638
Interventional	BMSC	Stem Cell Ophthalmology Treatment Study II (SCOTS2)	NCT03011541
Interventional	MSC	Safety of Cultured Allogeneic Adult Umbilical Cord Derived Mesenchymal Stem Cells for Eye Diseases	NCT05147701
Interventional	Idebenone	Raxone Treatment for Patients With Dominant Optic Atrophy due to OPA1 Gene Variant	EudraCT 2019-001493-28
Interventional	Idebenone	Idebenone Versus Placebo in Dominant Optic Atrophy	ACTRN12621000826842

Ongoing clinical trials as of August 2022. The following clinical trial registries were searched for “Dominant Optic Atrophy”: ClinicalTrials.gov (https://www.clinicaltrials.gov/), EU Clinical Trials Register (https://www.clinicaltrialsregister.eu/ctr-search/search) and International Clinical Trials Registry Platform (ICTRP; https://trialsearch.who.int/).

BMSC, bone marrow–derived stem cells; MSC, mesenchymal stem cells.

Gene therapies have also been developed to deliver neuroprotective factors like brain-derived neurotrophic factor (BNDF) (97). However, as most cases of DOA are caused by *OPA1* variants, efforts to develop gene therapies have focused on targeting the haploinsufficiency and dominant-negative mechanisms contributing to RGC loss. To address haploinsufficiency, gene replacement therapy has been explored in a mouse model. This involved intravitreal injections of an adenoassociated virus (AAV) vector carrying human *OPA1* cDNA into *Opa1*^*+/delTTAG*^ mice (98). Although this showed some protection of RGCs from degeneration, the method did not provide a way to control the level of *OPA1* expression. This is a concern because *OPA1* overexpression may be deleterious to cell function (64). The eye is uniquely advantaged in the field of gene therapy because it is immunologically privileged and has a relatively easy route of delivery through intravitreal injection. Voretigene neparvovec (Luxturna) is an AAV-delivered gene therapy that is administered by subretinal injection to replace the mutant *RPE65* gene and halt retinal degeneration (99). In 2017, voretigene neparvovec was the first in vivo gene therapy to be approved by the US Food and Drug Administration. In addition, encouraging results have been seen in gene replacement therapy trials for LHON (100,101).

Another gene augmentation strategy involving artificial transcription factors (ATFs) may provide an alternative method to boost the expression of the normal *OPA1* allele. ATFs are proteins composed of a specific DNA-binding domain and a transcriptionally active domain that is predesigned to modulate transcription (102). ATFs and similar RNA-based strategies can target various aspects of transcription. In a recent in vitro study, engineered RNA-based splicing factors were used to modulate splicing in the setting of the c.1065+5G>A variant resulting in increased OPA1 expression levels (103).

Artificial expression of WT *OPA1* could be combined with a targeted knockdown of mutant transcripts in the setting of dominant negative variants. For example, small interfering RNAs (siRNAs) can be used to target specific RNA sequences, which leads to their degradation by means of the RNA interference pathway (104). Antisense oligonucleotides (ASOs/AONs) are smaller molecules usually made from 8 to 50 nucleotides that can also be used to target specific mRNAs for degradation, cause exon/pseudoexon skipping, and other forms of altering transcription (105–108). This strategy has shown potential in preclinical studies for cystic fibrosis (109), and some groups are developing ASOs for DOA (110). However, treatments involving siRNA or ASO administration involve repeated dosing to maintain a therapeutic level.

By contrast, genome editing would be a one-off treatment to correct the disease-causing variant. For example, the use of CRISPR-Cas9 to edit the *OPA1* locus in RGCs may provide an opportunity to correct variants in vivo, thereby preventing further neurodegeneration. Using this approach, an improvement in DOA phenotype has been achieved in RGCs differentiated from iPSCs derived from patients carrying pathogenic *OPA1* variants (111). However, several challenges will need to be overcome to move this therapeutic strategy into clinical practice. First, the homology-directed repair mechanisms often used in CRISPR-Cas9 have a relatively low efficiency (112) meaning that in vivo use would likely only correct a small number of RGCs. In addition, the current packaging constraints of an AAV vector (∼4.7 kb) make the inclusion of all of the gene editing components required for CRISPR-Cas9 editing into a single vector challenging. Perhaps, the most important potential barrier to clinical translation is the risk of off-target effects, which have been reported as >50% in some experiments (113). Unintended off-target effects in oncogenes or cell repair genes present the greatest risk, and this must be mitigated by robust screening before human use. Despite these concerns, the eye does offer several unique opportunities in the field of genetic therapies as a result of its immune privilege and its ease of access with an intravitreal injection (78).

The above methods focus on preventing or halting degeneration of RGCs in DOA. Regenerative approaches aim to replace cells that have already died using specific cell types differentiated from patient-derived iPSCs, which minimizes the risk of immune rejection. However, there are several challenges to clinical translation of this technique, not only replicating the accurate projection of long RGC axons along the optic nerve toward the lateral geniculate nucleus (114) but also cell survival, dendrite extension, synapse formation with native cells, implanted cell function, and integration to the degree that they restore macroscopic retinotopic organization. In addition, there are risks of genomic instability, teratogenicity, and immunogenicity that need to be overcome before clinical application (115–117). Nevertheless, several ongoing clinical trials are testing potential therapies for DOA involving stem cell injections that may provide a local neuroprotective effect (Table 1).

## CONCLUSIONS

DOA caused by *OPA1* variants is the commonest inherited optic neuropathy in the general population, resulting in progressive blindness in children and young adults. The pathophysiological links connecting *OPA1* mutations with the peculiar selective vulnerability of RGCs have not yet been fully established, which has limited the therapeutic development for this disorder. Although the underlying pathogenesis has an heterogenous and often complex molecular basis, it is characterized by a final common pathway of mitochondrial dysfunction that is preferentially deleterious to RGCs, ultimately resulting in optic nerve degeneration and visual loss. Recent advances in our understanding of this process have been driven by a concurrent maturation of in silico, in vitro, and in vivo models, which is providing a wealth of exciting novel therapeutic targets, not only for DOA but also more broadly for other optic neuropathies and neurodegenerative diseases that are also thought to be driven, at least partly, by impaired mitochondrial biogenesis.
